# Human Newborn Monocytes Demonstrate Distinct BCG-Induced Primary and Trained Innate Cytokine Production and Metabolic Activation *In Vitro*

**DOI:** 10.3389/fimmu.2021.674334

**Published:** 2021-07-13

**Authors:** Asimenia Angelidou, Joann Diray-Arce, Maria-Giulia Conti, Mihai G. Netea, Bastiaan A. Blok, Mark Liu, Guzman Sanchez-Schmitz, Al Ozonoff, Simon D. van Haren, Ofer Levy

**Affiliations:** ^1^ Department of Neonatology, Beth Israel Deaconess Medical Center, Boston, MA, United States; ^2^ Precision Vaccines Program, Division of Infectious Diseases, Boston Children’s Hospital, Boston, MA, United States; ^3^ Department of Pediatrics, Harvard Medical School, Boston, MA, United States; ^4^ Department of Maternal and Child Health, Sapienza University of Rome, Rome, Italy; ^5^ Department of Internal Medicine and Radboud Center for Infectious Diseases, Radboud University Medical Center, Nijmegen, Netherlands; ^6^ Department for Genomics & Immunoregulation, Life and Medical Sciences Institute (LIMES), University of Bonn, Bonn, Germany; ^7^ Broad Institute of MIT & Harvard, Cambridge, MA, United States

**Keywords:** Bacille Calmette-Guérin (BCG) vaccine, cord blood, cytokines, lactate, newborn monocytes, immunometabolism, trained immunity

## Abstract

**Background:**

Newborns exhibit distinct immune responses and are at high risk of infection. Neonatal immunization with BCG, the live attenuated vaccine against tuberculosis (TB), is associated with broad protection against a range of unrelated pathogens, possibly reflecting vaccine-induced training of innate immune cells (“innate memory”). However, little is known regarding the impact of age on BCG-induced innate responses.

**Objective:**

Establish an age-specific human monocyte *in vitro* training platform to characterize and compare BCG-induced primary and memory cytokine responses and immunometabolic shifts.

**Design/Methods:**

Human neonatal and adult CD33-selected monocytes were stimulated for 24h with RPMI (control) or BCG (Danish strain) in 10% autologous serum, washed and cultured for 5 additional days, prior to re-stimulation with the TLR4 agonist LPS for another 24h. Supernatants were collected at Day 1 (D1) to measure *primary* innate responses and at Day 7 (D7) to assess *memory* innate responses by ELISA and multiplex cytokine and chemokine assays. Lactate, a signature metabolite increased during trained immunity, was measured by colorimetric assay.

**Results:**

Cytokine production by human monocytes differed significantly by age at D1 (primary, BCG 1:750 and 1:100 vol/vol, p<0.0001) and D7 (innate memory response, BCG 1:100 vol/vol, p<0.05). Compared to RPMI control, newborn monocytes demonstrated greater TNF (1:100, 1:10 vol/vol, p<0.01) and IL-12p40 (1:100 vol/vol, p<0.05) production than adult monocytes (1:100, p<0.05). At D7, while BCG-trained adult monocytes, as previously reported, demonstrated enhanced LPS-induced TNF production, BCG-trained newborn monocytes demonstrated tolerization, as evidenced by significantly diminished subsequent LPS-induced TNF (RPMI vs. BCG 1:10, p <0.01), IL-10 and CCL5 production (p<0.05). With the exception of IL-1RA production by newborn monocytes, BCG-induced monocyte production of D1 cytokines/chemokines was inversely correlated with D7 LPS-induced TNF in both age groups (p<0.0001). Compared to BCG-trained adult monocytes, newborn monocytes demonstrated markedly impaired BCG-induced production of lactate, a metabolite implicated in immune training in adults.

**Conclusions:**

BCG-induced human monocyte primary- and memory-innate cytokine responses were age-dependent and accompanied by distinct immunometabolic shifts that impact both glycolysis and training. Our results suggest that immune ontogeny may shape innate responses to live attenuated vaccines, suggesting age-specific approaches to leverage innate training for broad protection against infection.

## Introduction

As compared to other age groups, human newborns are highly susceptible to infections due in part to functionally distinct innate (1) and adaptive immunity (2). Epidemiologic studies have linked early life BCG immunization to an unanticipated reduction (~50%) in all-cause mortality, greatly exceeding that attributable to tuberculosis (TB) (3, 4). These observations suggest BCG induces heterologous protection against antigenically diverse, unrelated pathogens. One of the suggested mechanisms for heterologous protection against infection in the context of BCG vaccination is the novel concept of innate immune memory, also termed as “trained immunity” (5). Trained immunity is the ability of innate immune cells to mount an altered response against infection following a previous unrelated infection or vaccination.

Several lines of evidence suggest that trained immunity occurs in newborns (6). In mice, pre-treatment with Toll-like receptor (TLR) agonists enhances subsequent responses to polymicrobial sepsis (7) and treatment with BCG results in enhanced emergency granulopoiesis (8). Evidence that such trained immunity occurs in human newborns includes: (a) critically ill preterm newborns demonstrate enhanced pathogen-specific mononuclear cell pattern recognition receptor (PRR) expression in the setting of Gram-positive or Gram-negative bacteremia (9); and (b) histologic chorioamnionitis affecting preterm infants is associated with a significantly reduced risk of late onset bacterial sepsis (10). These observations suggest the existence of neonatal innate memory that alters responses to subsequent unrelated microbial challenges (11).

Early life immunization in Guinea-Bissau with BCG had beneficial effects on overall mortality, especially when provided at birth, with the largest effect seen in low birth weight newborns and during the first 2 months of life (12–14). In addition to reduced mortality, heterologous beneficial BCG effects in early life include reductions in respiratory infections and sepsis, in both high- and low-income settings (15, 16). *In vitro*, stimulation of BCG-trained adult peripheral blood mononuclear cells (PBMCs) with heterologous TLR agonists and bacteria led to increased production of TNF (17). BCG, given 1 month prior to an infectious challenge, enhanced clearance of yellow fever vaccine strain viremia, an effect that correlated with higher pro-inflammatory cytokine production (TNF, IL-1β, IL-6) from BCG-vaccinated adult volunteers, with a crucial role for IL-1β production (18). Such trained immunity effects have been ascribed to genome-wide epigenetic reprogramming of monocytes (Mos), which in adults is accompanied by metabolic rewiring, crucial for the induction of the histone modifications and functional changes underlying BCG-induced trained immunity in adults (19). Ongoing clinical trials are underway to assess BCG pathogen-agnostic protection against COVID-19 (20).

The extent, mechanism and ontogeny of trained immunity in early life remain incompletely defined. Understanding how BCG-induced innate immune engagement, including the enhancement of Th-polarizing cytokine production by antigen-presenting cells, varies by age, is of basic and translational importance (21, 22). In this study, we compared the impact of BCG stimulation on innate cytokine and chemokine responses by CD33+ monocytes characterizing both primary and memory innate responses of human newborn and adult monocytes to a subsequent stimulation with lipopolysaccharide (LPS). We found that in marked contrast to increased cytokine induction by BCG-trained adult monocytes, BCG-trained newborn monocytes mounted a tolerogenic response to endotoxin. Remarkably BCG-induced cytokines and chemokines at Day 1 inversely correlated with subsequent LPS-induced TNF production on Day 7. Moreover, human newborn monocytes failed to produce lactate in response to BCG, suggesting distinct immunometabolism in early life that could contribute to age-dependent effects of BCG.

## Methods

### Human Blood Collection

In accordance with approved protocols from the Institutional Review Board (IRB) of the Beth Israel Deaconess Medical Center, Boston, MA (protocol number 2011P-000118) and The Brigham & Women’s Hospital, Boston, MA (protocol number 2000-P-000117), human cord blood samples were collected from healthy full-term cesarean deliveries (>37 weeks gestational age). All de-identified blood samples from adult (age 18–40 years old) participants were collected with approval from the IRB of Boston Children’s Hospital, Boston, MA (protocol number 307-05-0223), after written informed consent. Small blood samples (10-15ml) were collected in vacutainer serum collection tubes (BD Biosciences; San Jose, CA) to secure autologous serum from each participant, and the remaining blood volume was anti-coagulated with 15 U/ml of pyrogen-free heparin sodium (Sagent Pharmaceuticals; Schaumburg, IL, USA) and assayed within 4h. Prior to study blood collection, none of the study participants had ever received BCG.

### Autologous Serum Preparation

Blood collected in vacutainer serum collection tubes was left undisturbed at room temperature for 30 min and allowed to clot, then centrifuged at 1500 × *g* for 10 minutes. Serum was maintained at 2-8°C during handling or apportioned in 0.5-1 ml aliquots and stored in -20°C until use.

### Isolation of Human Mononuclear Cells and Monocytes

Heparinized cord blood from newborns and peripheral blood from adults was centrifuged for 10 min at 500 × *g*, then the upper layer of clear yellow plasma was removed. The remaining blood was reconstituted to its original volume by resuspending in Dulbecco’s Phosphate Buffered Saline (DPBS, Life Technologies, Carlsbad, CA). Then, 25 ml of reconstituted blood was layered on to 15 ml of Ficoll-Hypaque gradients (Ficoll-Paque PREMIUM; GE Healthcare, Waukesha, WI) and centrifuged for 30 min at 500 × *g*. After Ficoll separation, the mononuclear cell fraction was collected. Monocytes were then isolated from mononuclear cell fractions by positive selection with magnetic CD33 MicroBeads, performed according to the manufacturer’s instructions (Miltenyi Biotec, Auburn, CA). CD33 *vs.* CD14 was chosen in order to avoid induction of non-physiological activation and provide more natural monocyte heterogeneity (23, 24). Purity was checked by flow cytometry and was always > 90%.

### Trained Immunity Assay

**Figure 1:**
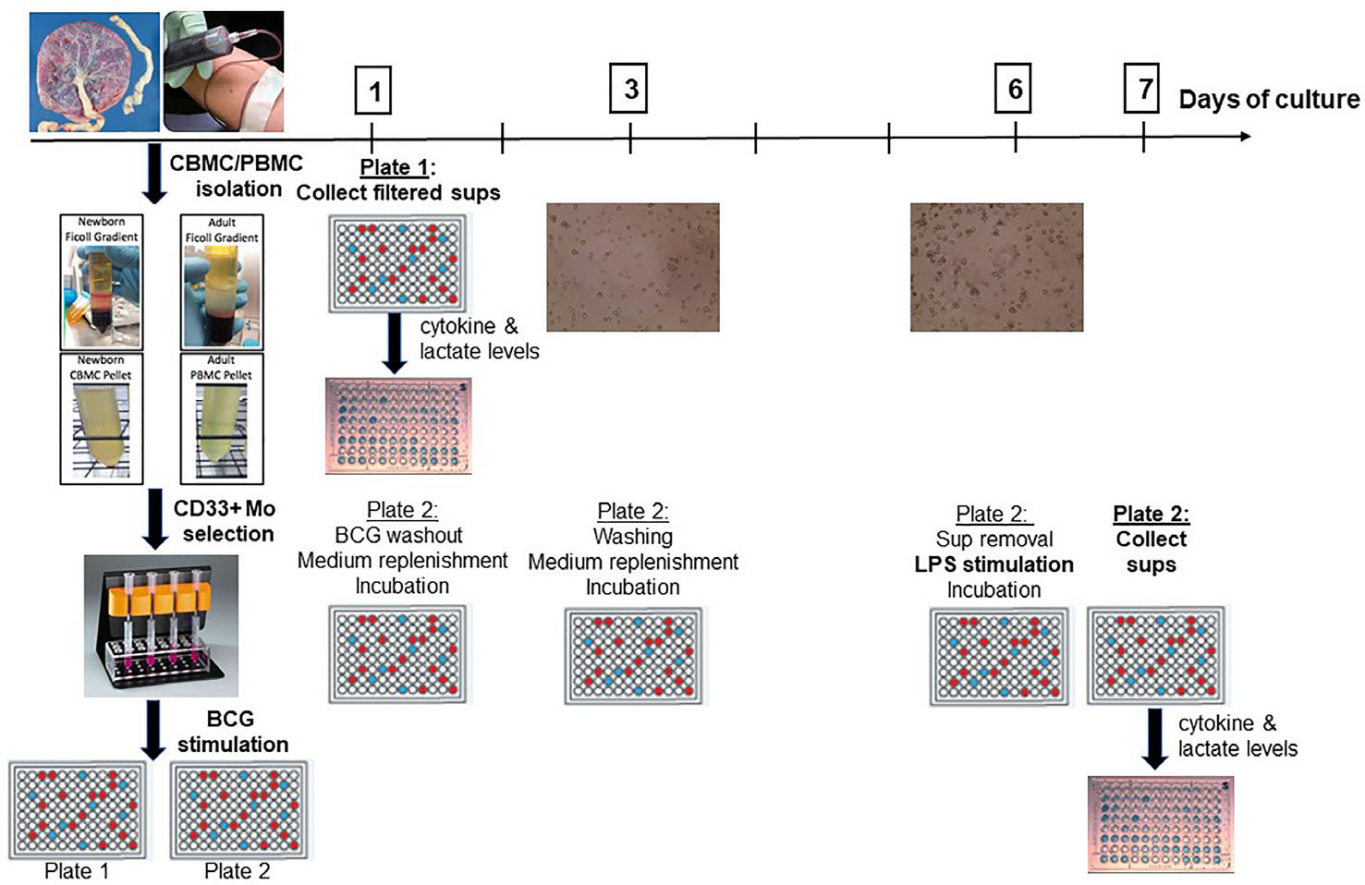
A human *in vitro* platform to assess age-dependent BCG training. Peripheral venous blood was drawn from healthy adult volunteers and cord blood was collected from healthy term (≥ 37 weeks gestation) elective cesarean deliveries. After PBMC and CBMC isolation, pure CD33+ Mos were isolated by immunomagnetic separation and plated in two separate 96-well plates. After 1h of resting, Mos were stimulated with control RPMI medium or with BCG. Supernatants were harvested from the first plate at 24h post-stimulation for Day 1 cytokine and lactate measurements. The second plate was further cultured after BCG was filtered out at 24h to allow time for immune training. After intermediate washing and culture medium replenishment steps, trained and untrained (control) monocytes were stimulated at Day 6 with LPS for 24h at which point supernatants were harvested for cytokine and lactate measurements. PBMC, peripheral blood mononuclear cells; CBMC, cord blood mononuclear cells; Mo, monocyte; sups, supernatants.

Following isolation, monocytes were counted and re-suspended in a Roswell Park Memorial Institute (RPMI) 1640 medium supplemented with L-glutamine, penicillin, streptomycin and 10% autologous serum at a concentration of 1 million monocytes/ml. After a 1h resting phase, monocytes were stimulated with BCG or RPMI (negative control) in duplicate: one singlicate was used to study primary innate responses and supernatants were filtered and harvested after 24h for cytokine measurements and lactate measurements. The other singlicate was used to study trained immunity: After 24h, BCG was washed out with a calcium- and magnesium-free PBS [PBS(−)], medium was replenished, and BCG-trained or control monocytes were incubated at 37°C for 5 days with an interim medium replenishing step on Day 3 of culture. On Day 6, supernatants were collected and a second stimulus, lipopolysaccharide from *Salmonella minnesota* (LPS-SM Ultrapure, Invivogen; San Diego, CA) was added. After 24 h incubation, supernatants were harvested on Day 7 of culture for cytokine and lactate measurements (**Figure 1**).

### Cytokine/Chemokine Assays

Supernatants from the trained immunity assay were analyzed for TNF with a human ELISA kit (BD Opteia ELISA set, BD Biosciences; San Jose, CA) as per the manufacturer’s directions. ELISA plates were read on a Versamax microplate reader with SoftMax Pro Version 5 (both from Molecular Devices; Sunnyville, CA). A fluorescent bead-based multianalyte xMAP technology cytokine kit (Milliplex Human Cytokine/Chemokine Immunoassay, Millipore Corp; Billerica, MA) was employed to measure the concentration of 41 analytes including Th1-, Th2-, and Th17- cytokines, chemokines and hematopoietic factors. Assays employed a Luminex 200 Bioanalyzer (Luminex Corp; TX, USA) set to acquire at least 50 events per cytokine. Multiplex cytokine/chemokine data were analyzed using *BeadView* multiplex Data Analysis Software (v.1), according to the manufacturer’s instructions (Millipore).

### Lactate Measurements

Lactate concentrations in culture supernatants were measured post-primary stimulation (Day 1) and post-secondary stimulation (Day 7) using a colorimetric assay (Lactate Kit II, Biovision; Milpitas, CA).

### Statistical Analysis

Cytokine/chemokine concentrations were normalized to RPMI control, log_2_-transformed and represent log-fold-change over RPMI. To assess statistical significance, differences between individual treatment conditions (for example BCG 1:10 vs RPMI) were evaluated by Student’s t-test, while differences between age groups and across BCG concentrations were evaluated by ANOVA.

Lactate production was normalized to the vehicle condition on Day 1 and log_2_-transformed. The paired Student’s t-test was used for comparison of trained *vs.* untrained conditions and within each age group over time. Unpaired Student’s t-test was used for comparison of similar conditions between age groups.

We used Pearson correlation to evaluate associations between primary cytokine/chemokine concentrations (Day 1) and subsequent LPS-induced TNF trained immune responses (Day 7). A *p*-value <0.05 was considered statistically significant. Statistical and graphical analysis was performed using *Prism* 7 software (Graph Pad Software Inc; La Jolla, CA), R version 4.0 and geepack v 1.3-2 package.

## Results

A human neonatal *in vitro* trained immunity platform was designed based on studies of adult monocyte BCG-induced trained immunity *in vitro* assays, which were used as a benchmark (25). In accordance with these studies, we employed LPS, a TLR4 agonist which is not present on the mycobacterial surface, as our heterologous stimulus of choice. We defined Day 0 as the day of blood collection and primary monocyte stimulation. We confirmed that assessment of a trained immunity response was optimal after 7 days of culture and used this as our timepoint of choice.

**Figure 2:**
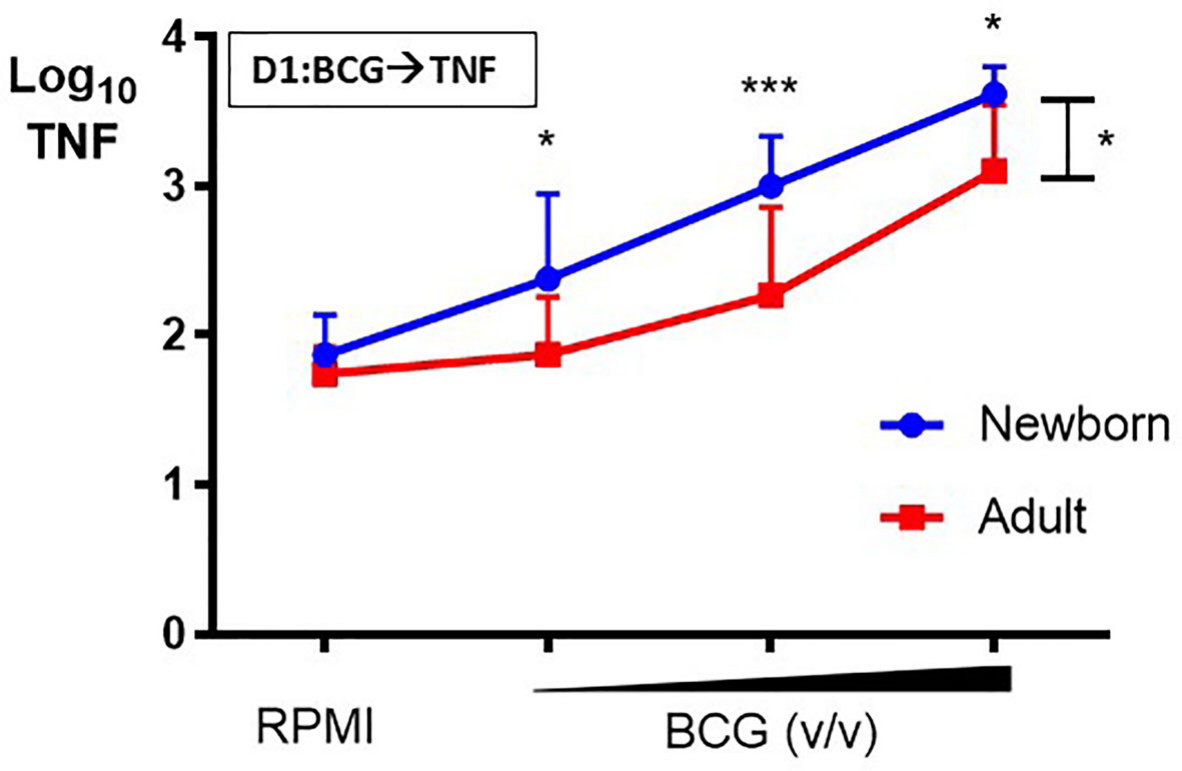
Human newborn monocytes demonstrate distinct BCG-induced primary TNF responses. Human newborn and adult CD33+ monocytes were cultured *in vitro* as described in **Figure 1**. In contrast to adult monocytes, neonatal monocytes demonstrated relatively greater primary TNF responses to BCG. Results are shown as log_10_ cytokine concentrations due to skewed distribution of values. N = 7 newborns, 9 adults. D, Day; v/v, volumetric concentrations. Bars indicate mean + SD. Repeated-measures 1-way ANOVA was used for comparisons across BCG concentrations and 2-way ANOVA was used for comparisons between age groups. *p < 0.05; ***p < 0.001.

Primary BCG-induced TNF production by human CD33+ Mos isolated from adult peripheral and cord blood mononuclear cells was assessed after *in vitro* stimulation. Newborn Mos stimulated with increasing concentrations (vol/vol 1:750, 1:100, 1:10) of BCG- Denmark generated concentration-dependent increases in TNF production compared to control RPMI medium (RM 1-way ANOVA, ***p=0.0002; **Figure 2**). Although adult Mos also exhibited concentration-dependent increases in TNF production compared to control RPMI medium (RM 1-way ANOVA, *p=0.01), they produced significantly lower BCG-induced TNF compared to newborn Mos for each BCG concentration and overall (2-way ANOVA, *p=0.01) (**Figure 2**). Absolute TNF concentrations are shown in **Supplementary Figure 1**. Additional analyses fitting a linear trend showed that TNF values increase 80% on average for each step up in concentration among newborns (p<0.0001) vs. 56% increase for each step up in concentration among adults (interaction p=0.02). Overall, adult Mos produce 56% lower TNF on average compared to newborn Mos (p=0.002).

**Figure 3:**
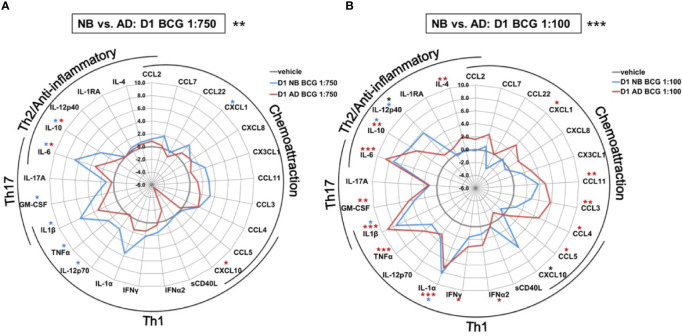
Human newborn and adult monocytes demonstrate distinct primary BCG-induced cytokine and chemokine production at Day 1 of culture. Human newborn and adult CD33+ monocytes were cultured *in vitro* as described in **Figure 1** then stimulated for 24 hours with **(A)** low (1:750 vol/vol) or **(B)** high (1:100 vol/vol) concentrations of BCG prior to measurement of cytokine and chemokine production in supernatants using a multiplex assay as described in *Methods*. Data was normalized to RPMI control, log_2_ transformed, and represents log_2_ fold-change. N = 5 newborns and 7 adults; *p < 0.05; **p < 0.01; ***p < 0.001 (blue stars: NB *vs.* vehicle; red stars: AD *vs.* vehicle; black stars: NB *vs.* AD). Vehicle-(re)stimulated conditions shown in gray.

The effect of BCG on production of a range of cytokines and chemokines was further examined in human Mos stimulated with a low or high BCG concentration- 1:750 (vol/vol) and 1:100 (vol/vol), respectively (**Supplementary Table 1**). Primary (24h after BCG stimulation) BCG-induced Mo cytokine/chemokine production significantly differed by age (RM-ANOVA, BCG 1:750, p<0.01; BCG 1:100, p <0.001). Specifically, after stimulation with the low BCG concentration, newborn Mos demonstrated higher log-fold increases for the vast majority of cytokine/chemokine concentrations as compared to RPMI than adult Mos (**Figure 3A**). Cytokines that were significantly elevated in newborn Mos compared to RPMI included IL-1β, TNF, IL-12p70, IL-6 and IL-10 (**Supplementary Figure 2**).

In contrast to neonatal Mos, adult Mos demonstrated higher log-fold increases for the vast majority of cytokine/chemokines as compared to RPMI than newborn Mos after stimulation with the high BCG concentration (**Figure 3B**). Cytokines that were significantly elevated compared to RPMI in adult Mos included TNF, IFNγ, IFNα2 and IL-4. Several chemoattractants were also significantly elevated in the adult Mos only, such as CXCL1, CCL11 (formerly eotaxin-1), CCL3 and CCL4. At the high BCG dose, the age-differential effect of BCG was particularly evident for IL-12p40 and CXCL10 with the same or opposite directionality of production in newborns and adults, respectively.

**Figure 4:**
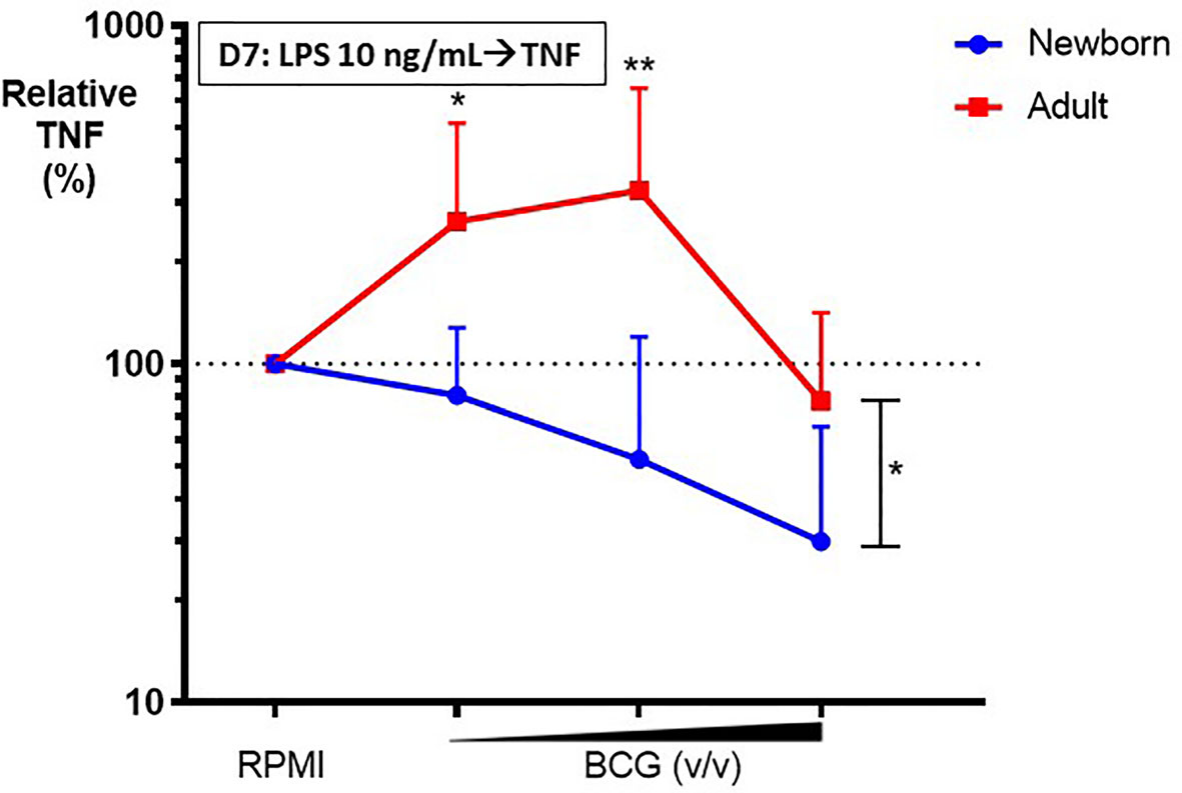
BCG-training induces enhanced LPS-induced TNF responses in adult monocytes but diminished TNF responses in newborn monocytes. Human newborn and adult CD33+ monocytes were trained with BCG as described in **Figure 1**. At Day 7 of culture, monocytes were stimulated by LPS prior to collection of supernatants for TNF ELISA. Relative TNF is the calculated ratio of trained vs. untrained TNF concentrations. N = 7 newborns, 9 adults. D, Day; v/v, volumetric concentrations. Bars indicate mean + SD. Repeated-measures 1-way ANOVA was used for comparisons across BCG concentrations and 2-way ANOVA was used for comparisons between age groups. *p < 0.05; **p < 0.01.

To assess the effects of BCG on Mo innate immune memory, control (RPMI)- or BCG- stimulated human Mos underwent a procedure to filter out BCG at 24h post-stimulation (as described in the *Methods* section) and were then cultured in parallel for 5 more days to allow for *in vitro* BCG training. At Day 6 of culture, control and BCG-trained Mos were stimulated with RPMI (to allow for background correction of TNF concentrations at Day 6 of culture) or a heterologous stimulus, LPS, and resulting TNF production was measured 24h later at Day 7 of culture. Compared to the control RPMI-trained adult Mos, BCG-trained adult Mos demonstrated enhancement of subsequent LPS-induced TNF production for 2 of the 3 BCG concentrations tested (**Figure 4**). Remarkably, in contrast, BCG-trained newborn Mos demonstrated a dose dependent *decrease* in LPS-induced TNF production compared to the RPMI-trained (control) newborn Mos for all BCG concentrations tested. The effect of BCG-training on TNF responses was significantly different for 2 of the 3 BCG concentrations tested (BCG 1:750 and BCG 1:100 v/v) in newborn *vs.* adult Mos and had the opposite direction between age groups (**Figure 4**). Decrease in TNF concentration at the highest BCG concentration (BCG 1:10 v/v) was accompanied by a relative loss of Mo viability, which was of similar magnitude in both age groups (**Supplementary Figure 3** **).** Limited immunophenotyping assessment of Mo cell surface markers CD14, CD11b and TLR4 by flow cytometry raised the possibility of distinct BCG-induced phenotypic changes between newborn and adult Mos (data not shown), but further study of this phenomenon will be required given the limited nature of this data set. Notably, vaccination of adult study participants with BCG *in vivo* was associated with a decrease in Mo TLR4 expression 2 weeks post-immunization (17), suggesting early immunophenotypic changes in trained immunity.

**Figure 5:**
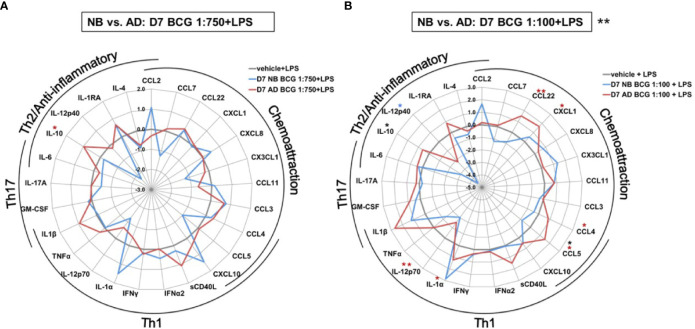
Distinct LPS-induced cytokine production by BCG-trained human newborn vs. adult monocytes at Day 7 of culture. Human newborn and adult CD33+ monocytes were cultured *in vitro* as described in **Figure 1**. Cytokines and chemokines were measured by Multiplex assay. Data shown for BCG concentrations **(A)** 1:750 vol/vol and **(B)** 1:100 vol/vol. Data was normalized to RPMI control, log_2_ transformed, and represents log_2_ fold-change. N = 5 newborns and 7 adults; *p < 0.05; **p < 0.01 (blue stars: NB *vs.* vehicle; red stars: AD *vs.* vehicle; black stars: NB *vs.* AD). Vehicle-(re)stimulated conditions shown in gray.

In addition to the aforementioned differences in TNF production between newborns and adults the impact of BCG training on a variety of cytokine responses also differed markedly by age (**Figure 5** and **Supplementary Table 2**). Effects were significantly different between newborns and adults after priming with the high BCG dose and restimulation with LPS [BCG 1:100; RM-ANOVA, p=0.01]; **Figure 5B**). IL-10 concentrations were not only significantly different, but also divergent between newborns and adults with a recorded 5 log_2_-fold decrease in newborn IL-10 concentrations relative to RPMI control. The directionality of change in newborns was opposite from that in adults and statistically significant for CCL5. Production of IL-12p40, which was significantly upregulated by primary BCG stimulation, was significantly attenuated by LPS restimulation of BCG pre-exposed newborn Mos.

**Figure 6:**
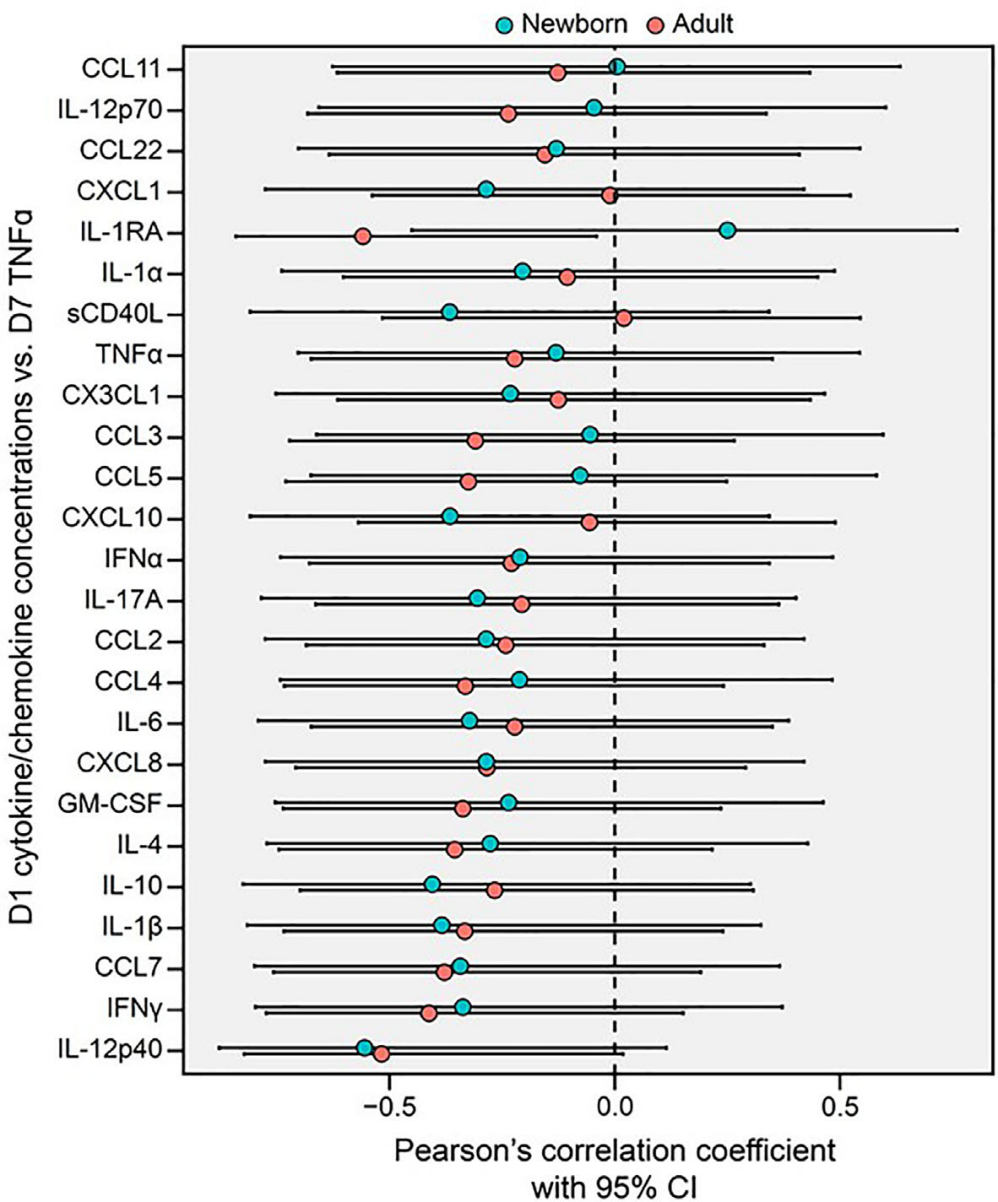
Primary BCG-induced cytokine/chemokine concentrations (Day 1, D1) of human monocytes inversely correlate with their trained TNF cytokine concentrations (Day 7, D7) in newborns and adults. A Forest plot depicts pairwise comparisons between newborn and adult D1 cytokine/chemokine *vs.* D7 TNF correlations. Correlations between the cytokine and chemokine data depicted in **Figures 3**, **5** were quantified using Pearson’s coefficient. Error bars represent the associated 95% CI.

We next assessed whether BCG-induced Mo primary cytokine/chemokine responses on Day 1 correlated with subsequent trained LPS-induced TNF responses at Day 7. BCG-induced adult Mo production of IL-1RA on Day 1 inversely correlated with subsequent LPS-induced TNF on Day 7 (R=-0.56, *p=0.04), while newborn Mos demonstrated a positive correlation for this cytokine (R +0.25) (**Figure 6****).** Using the signed rank test, correlation coefficients of Day 1 cytokines/chemokines and subsequent Day 7 LPS-induced TNF production were generally negative in both age groups (p<0.001 for both age groups; **Supplementary Figure 4**).

**Figure 7:**
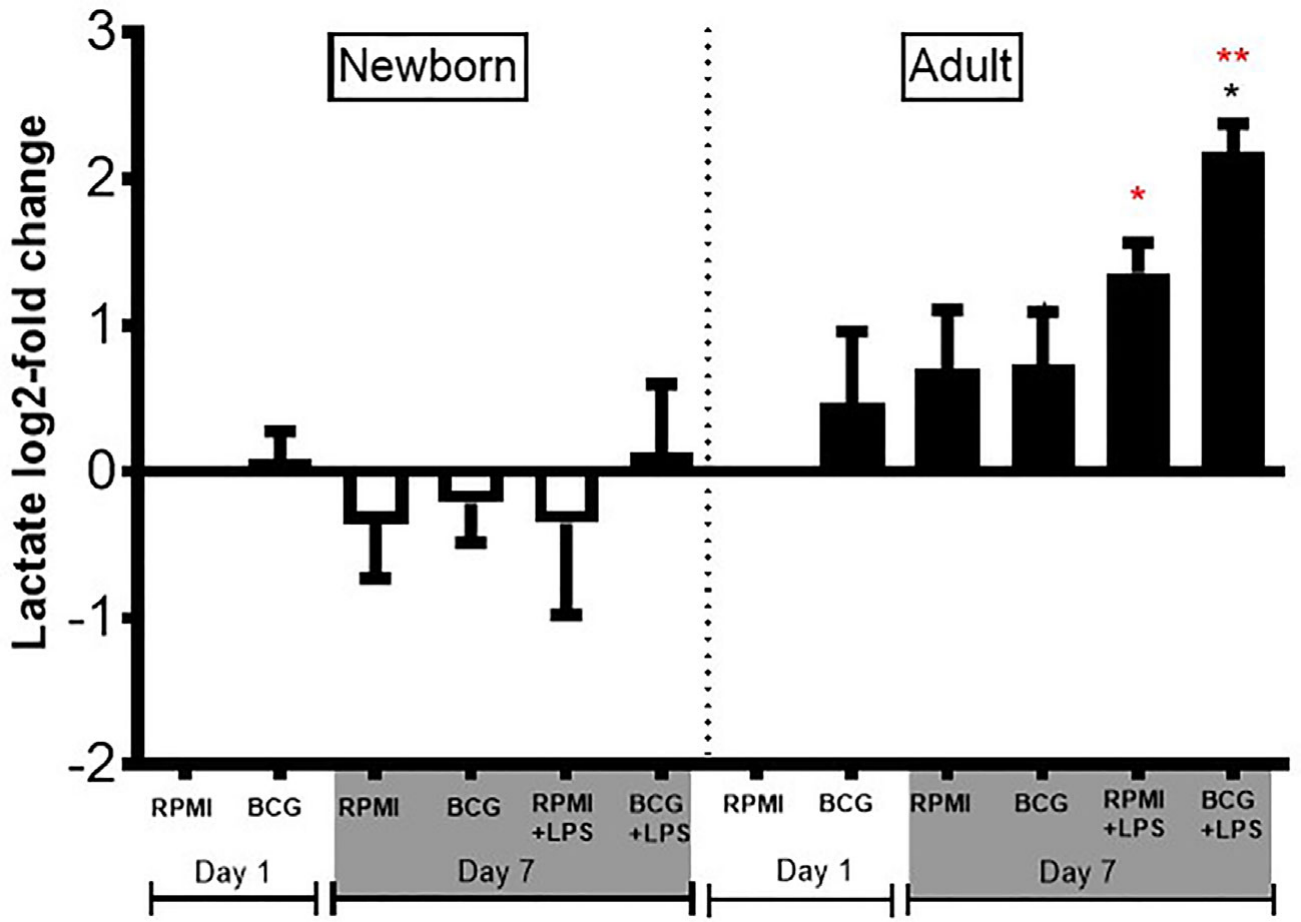
Diminished lactate production in human newborn vs. adult monocytes. Human newborn and adult CD33+ monocytes were cultured *in vitro* as described in **Figure 1**. Lactate was measured in culture supernatants post-primary BCG stimulation (Day 1) and post-secondary LPS stimulation (Day 7) using a colorimetric assay as described in *Methods*. Lactate production was normalized to the vehicle condition on Day 1 and log_2_-transformed. N = 4 newborns and 4 adults. Bars indicate mean + SD. *p ≤ 0.05, **p ≤ 0.01 (black star: log_2_-fold change compared to untrained control RPMI+LPS at Day 7; red stars: NB *vs.* AD for the respective conditions).

As newborns have distinct immunobiology, immunity, and metabolism (26, 27), as well as BCG-induced primary and trained cytokine production, we hypothesized that BCG priming may have distinct immunometabolic effects towards newborn vs adult Mos. To test this hypothesis, we measured lactate production in supernatants from our Mo training assay. BCG-trained adult Mos subsequently treated with LPS produced 2 to 3 log_2_-fold more lactate compared to RPMI control (**Figure 7**), as expected given that glycolysis is associated with innate immune activation in adults (28). In marked contrast to their adult counterparts, newborn Mos did not exhibit any significant increase in lactate from baseline but rather trended towards diminished lactate concentrations, suggesting absence of a metabolic switch toward increased glycolysis (**Figure 7**).

## Discussion

While there is growing evidence that BCG re-shapes innate immune responses to tuberculosis-unrelated pathogens potentially accounting for pathogen- agnostic protection and clinical benefit, the underlying mechanisms for these heterologous effects in early life are incompletely characterized (29). Herein, we demonstrate for the first time that BCG has distinct age-specific effects on human newborn Mos, including distinct primary innate cytokine responses as well as trained immunity.

To characterize age-specific effects of BCG, we utilized a human *in vitro* trained immunity platform using primary human Mos cultured in autologous serum to compare newborn and adult Mo primary and trained responses to BCG. We view the use of autologous plasma or serum (intact, i.e. not heat-treated and from the same individual), repleted with age-specific immune factors such as maternal antibodies, adenosine and prostaglandins (30), as an important element of our design which strives to remain faithful to physiologic conditions that are relevant *in vivo*.

Compared to their adult counterparts, human newborn Mos responded to BCG in a fundamentally distinct manner. With respect to the primary response to BCG, newborns responded more robustly to the low concentration (1:750 vol/vol) relative to RPMI, as demonstrated by significantly enhanced production of Th1 (IL-12p70 and TNF), Th2 (IL-6 and IL-10) and Th17- (IL-1ß and GM-CSF) polarizing cytokines, as well as of the chemoattractant CXCL1. A possible explanation for the enhanced response of newborn Mos to low-dose BCG compared to adult Mos could be age-dependent differences in the magnitude of TLR responses (31–33), bearing in mind that BCG activates TLR8 (34) that is a power activating pathway in the newborn (35). The complex nature of BCG as a live mycobacterial stimulus that activates multiple PRRs (36) likely explains primary production of broadly acting cytokines. IL-12p70 induces cytotoxic T cell responses as well as high and broad humoral immune responses (37). TNF concentrations *in vitro* have been used as a benchmark cytokine for BCG-trained immunity in adults (17), while IL-1β production is implicated in BCG-trained innate immunity in adults and low birth weight infants (4, 38). GM-CSF may contribute to the host response against mycobacterial infection by favoring macrophage M1 polarization after *Mycobacterium bovis* BCG infection (39), as well as regulating the neutrophil-mediated inflammatory response, which mediates BCG-induced protection in a mouse model of neonatal polymicrobial sepsis (8).

Adults overall responded more robustly to the higher concentration of BCG (1:100 vol/vol), and specifically exceeded neonatal responses in production of IL-12p40 and CXCL10 (formerly IP10). In addition to its chemotactic properties, CXCL10 is also involved in the stimulation of natural killer and T-cell migration in response to *Mycobacterium tuberculosis* infection (40). Selective induction of the IL-12p40 component of the IL-12 cytokine and subsequent development of T-follicular helper cells in the lymph node *via* upregulated IL-12-receptor signaling is a unique feature of live vaccines. Such BCG-induced IL-12 pathway activation is mediated *via* sensing of viability by TLR8 whose functional alleles correlate with protection vs. pulmonary TB in BCG-immunized adults, and is not observed with killed vaccines (34).

With respect to BCG-trained Mo responses to subsequent stimulation with LPS, neonatal Mos demonstrated a distinct profile. The directionality and magnitude of cytokine production of BCG-primed/LPS-restimulated newborn Mos was BCG-concentration dependent. Specifically, unlike BCG-trained adult Mos that demonstrated enhanced LPS-induced cytokine and chemokine production, BCG-trained newborn Mos displayed decreased LPS-induced TNF production. Multiplex analysis revealed that at the low concentration (1:750 vol/vol), BCG-trained adult Mos demonstrated greater LPS-induced IL-10 production. In contrast, at the high BCG concentration (1:100 vol/vol), BCG-trained newborn Mos demonstrated diminished LPS-induced IL-10 and CCL5 production compared to adult Mos, and significantly decreased IL-12p40 production compared to RPMI control-treated Mos. A similar tolerogenic response has been previously reported in whole blood of BCG-vaccinated infants, who demonstrated increased production of IFN-γ in response to mycobacterial stimulation, but decreased production of IFN-γ in response to subsequent heterologous stimulation and TLR agonists, as compared to BCG-naïve infants (41).

Correlations between BCG-induced human Mo production of individual cytokines/chemokines on Day 1 and subsequent LPS-induced TNF production on Day 7, could serve as novel cytokine/chemokine biomarker signatures of BCG-induced training in adults and tolerance in newborns. We found a significant moderate negative correlation in adults between primary IL-1RA and trained TNF production, while this correlation was positive in newborns. As IL-1β has an established role in trained immunity in adults (38), and IL-1RA may prevent IL-1β binding to its receptor, the interplay between these two cytokines in trained immunity and in particular their role in neonatal trained immunity is worthy of further exploration. In neonates, BCG vaccine induces production of IL-12, the primary cytokine that drives CD4+ T cell Th1 differentiation, in a TLR2-dependent manner (42). Our results indicate that early interaction of BCG with Mos shapes their subsequent responses to LPS, a heterologous innate stimulus and raises the possibility that the cytokines induced early may engage counter-regulatory pathways.

Overall, to the extent that our *in vitro* results are relevant *in vivo*, our observations suggest that BCG-induced trained immunity in the neonate may attenuate an overwhelming inflammatory response to potentially noxious subsequent stimuli. With respect to TNF production, after the initial phase of primary innate immune activation resulting in increased production and enhanced chemotaxis, BCG appears to re-wire neonatal Mos for a tolerogenic response to subsequent stimulation, as occurs in bacterial sepsis. However, the immune system likely seeks homeostasis and one way to prevent immunoparalysis, the extreme version of immune tolerance, is through concomitant decreased production of anti-inflammatory cytokines such as IL-10 and IL-12p40. Concurrently, decreased chemotaxis and angiogenesis may prevent untoward inflammatory sequelae. The overall pattern of BCG-trained Mo cytokine and chemokine responses suggests that neonatal Mo responses may serve a different purpose compared to adult Mo responses, where training seems to cause Th1 polarization of the innate immune response and enhancement of the inflammatory response.

A prior *in vitro* study comparing human cord blood Mos and adult peripheral blood Mos suggested similar cytokine production after BCG priming and LPS restimulation (43). Multiple differences in study design between our study and the prior one could account for our distinct findings, including a different study population (US-based cohort in our study *vs.* Norwegian in the prior study), use of different BCG formulations (BCG-Denmark in our study *vs.* BCG-Bulgaria in the prior), blood collection from BCG-naïve adult study participants in our study *vs.* previously BCG-immunized in the prior, the use of heparin *vs.* citrate (a calcium chelator) for blood collection, the use of untreated autologous newborn *vs.* adult serum in our study vs. 10% pooled sterile serum from humans of undescribed age in the prior study, which could obscure soluble plasma-based ontogenic differences that shape immune responses (30), and the method for Mo isolation (isolation of Mos by gradient-centrifugation in our study vs. adherence, which is an activating step, in the prior study). Different licensed BCG formulations vary substantially in their immune-stimulating capacity, including in induction of IL-1β, a cytokine key to trained immunity (38), correlating with differences in viability (44). This is especially notable in light of growing literature regarding differences between BCG vaccine formulations/strains in effectiveness in preventing tuberculosis and unrelated infections (45), with BCG-Denmark being the most frequently studied formulation for its trained immunity inducing properties *in vivo* and *in vitro* (29). Overall these multiple differences in study design, could have contributed to the prior study not demonstrating differences in cytokine production between newborns and adults.

Upon training with BCG and subsequent stimulation with LPS, whereas adult Mos demonstrated robust ~2 to 3 log_2_-fold higher lactate production compared to RPMI, newborn Mos demonstrated little lactate production, almost comparable to vehicle control (RPMI). Similar age-dependent immunometabolic differences were recently observed in another study of activated human cord blood and adult macrophages (46). Of note, mean lactate production of neonatal Mos at baseline is slightly lower than that of adult Mos and directly correlated with pyruvate kinase activity, which is diminished in newborn vs. adult Mos but reaches adult levels halfway through infancy (47). Overall, these observations collectively suggest that glycolytic metabolism of newborn Mos differs from that of adult Mos, possibly contributing to the distinct age-specific BCG-induced Mo training. Global metabolomic profiling of human newborn Mos may provide immunometabolic signals unique to BCG-trained immunity (48).

To the extent our *in vitro* results reflect the effects of BCG *in vivo*, the protective effects of BCG may in part rely on attenuating inflammatory responses to microbial products that signal *via* PRRs. The induction of training or tolerance appears to be dependent on the type and quantity of the microbial stimulus and host factors. BCG is a live and complex microbial stimulus that activates multiple PRRs, including TLRs (49–51), C-type lectin receptors (CLRs) (52), and NOD-like receptors (NLRs) (17). BCG training of adult Mos was associated with NOD-2-dependent epigenetic reprogramming (17). Among the PRRs, TLRs appear to play a prominent role in neonatal responses to immunization/infection as suggested by: a) an association between TLR polymorphisms and altered responses to neonatal BCG immunization (53), and b) selective predisposition to bacterial infection in young but not older children with genetic defects affecting TLR downstream signaling (IRAK-4, MyD88) (54). Of note, a microbial stimulus can elicit different responses when engaging different receptors. For example, LPS induces immunosuppressive effects when engaging TLRs vs. immunopotentiating effects when engaging NLRs (55). Innate immune memory responses are complex and depend on the age of the exposed, timing of exposure and properties of the stimulus. Of note, while neonatal Mos express similar quantities of TLRs as their adult counterparts (55, 56) the downstream consequences of TLR activation are distinct with age (8). Whether the BCG-induced tolerance to LPS in neonatal Mos observed in our study is related to TLR-mediated epigenetic reprogramming will be an important area of future investigation.

Our study features multiple strengths, including (a) use of species (human)- and age (newborn)-specific Mos cultured in autologous serum, (b) Mo-selection through CD33 instead of CD14 to avoid activation, (c) study of a licensed WHO-prequalified BCG formulation/strain, (d) assessment of BCG concentration-dependent effects, (e) study of both primary (24-hour stimulation) and trained (LPS-induced cytokine production at Day 7) immune effects, and (f) measurement of metabolic activation in the form of lactate production.

As with any research, our study also has some limitations including (a) an *in vitro* approach that likely does not capture all of the immunologic effects of BCG *in vivo* (17), (b) an exclusive focus on myeloid CD33+ mononuclear cells which, although important to BCG responses *in vitro* and *in vivo* (51), will not capture the full range of relevant human leukocyte responses to this live vaccine, and (c) a focus on LPS as a secondary stimulus which may not reflect responses to other PRR agonists. Spontaneous *in vitro* differentiation of Mos over time towards macrophage phenotypes is possible and has been previously described in culture medium supplemented with autologous serum (57). Detailed immunophenotyping assessment of BCG-treated human newborn and adult Mos should be pursued in future studies to provide a fuller picture of BCG’s age-dependent effects. Given the marked variability between BCG formulations/strains (44), future studies should also directly compare and characterize the impact of a range of BCG formulations/strains on the subsequent responses of a range of human leukocytes and innate stimuli.

In summary, BCG-induced training of human Mos is age-dependent, suggesting that immune ontogeny may shape primary and trained innate cytokine responses to BCG. Much remains to be learned about alterations in neonatal immune function following infection/vaccination during this critical period of immune system adaptation and development. Using BCG as a model to characterize distinct trained immunity in newborns may inform discovery and development of novel adjuvants, vaccines and immunotherapies for this vulnerable population (56).

## Data Availability Statement

The datasets presented in this study can be found in online repositories. The names of the repository/repositories and accession number(s) can be found below: Raw data files for cytokines, chemokines and lactate concentrations were deposited in ImmPort under accession number: SDY1790.

## Ethics Statement

The studies involving human participants were reviewed and approved by Institutional Review Board (IRB) of the Beth Israel Deaconess Medical Center, The Brigham & Women’s Hospital, Boston, MA and Boston Children’s Hospital, Boston, MA. Written informed consent for participation was not required for this study in accordance with the national legislation and the institutional requirements.

## Author Contributions

AA was the project lead and wrote the manuscript draft. AA, MGC, ML, and SvH collected and processed the samples and generated the data. MGN and BAB assisted with establishment of the trained immunity assay. AA, JD-A and AO analyzed the data. AA, SvH and OL interpreted the results. GS-S provided key intellectual input and edited the manuscript. All authors contributed to the article and approved the submitted version.

## Funding

This work was supported in part by grants from the National Institute of Allergy and Infectious Diseases (NIAID), including U01AI124284 to SvH and a Human Immunology Project Consortium (HIPC) award (5U19AI118608) to OL as well as the Mueller Health Foundation. AA received the Marshall Klaus Perinatal Research Award from the American Academy of Pediatrics. MGC was sponsored by Sapienza University of Rome and was a recipient of the *Admeto Pettinnari e Paolo Andreini* graduate scholarship for specialization courses in Italy and abroad. MGN was supported by a Spinoza grant of the Netherlands Organization for Scientific Research. AO, GSS, OL, and SvH were in part supported by the *Precision Vaccines Program* at Boston Children’s Hospital. The *Precision Vaccines Program* is supported in part by the BCH Department of Pediatrics and the Chief Scientific Office.

## Conflict of Interest

OL is a named inventor on vaccine adjuvant patent applications as well as an issued patent on an *in vitro* microphysiologic tissue construct platform for vaccine evaluation.

The remaining authors declare that the research was conducted in the absence of any commercial or financial relationships that could be construed as a potential conflict of interest.

## Abbreviations

BCG, Bacille Calmette-Guérin; CBMC, cord blood mononuclear cells; DPBS, Dulbecco’s Phosphate Buffered Saline; LPS-SM, Lipopolysaccharide from *Salmonella Minnesota*; Mo, monocytes; PBMC, peripheral blood mononuclear cells; PRR, pattern recognition receptors; RPMI, Roswell Park Memorial Institute; TB, tuberculosis; TLR, toll-like receptor.
