# Supplementary material for: Human Newborn Monocytes Demonstrate Distinct BCG-Induced Primary and Trained Innate Cytokine Production and Metabolic Activation In Vitro
Source: Front Immunol. 2021 Jul 13;12:674334. doi: 10.3389/fimmu.2021.674334 (PMC8315003; doi:10.3389/fimmu.2021.674334)
Supplement: Supplementary file 1 [file DataSheet_1.docx]

Supplementary Material

**Supplementary Figure 1:** **Human newborn monocytes demonstrate distinct BCG-induced primary TNF responses.** Human newborn (NB) and adult (AD) CD33+ monocytes were cultured *in vitro* as described in Figure 1. In contrast to adult monocytes, neonatal monocytes demonstrated relatively greater primary TNF responses to BCG. Absolute TNF concentrations (pg/mL) are shown. N= 7 newborns, 9 adults. v/v: volumetric concentrations. Bars indicate mean + SD. Repeated-measures 1-way ANOVA was used for comparisons across BCG concentrations with follow-up Fisher’s LSD testing for each BCG concentration vs RPMI control. Two-way ANOVA was used for comparisons between age groups. ** p<0.05; ** p<0.01; *** p<0.001; **** p<0.0001.* (blue stars: NB vs. vehicle; red stars: AD vs. vehicle; **black stars:** NB vs. AD).

**Supplementary Figure 2:** Absolute concentrations (pg/mL) of select cytokines after stimulation for 24 hours with increasing BCG concentrations (RPMI without BCG, low BCG concentration (1:750 vol/vol), high BCG concentration (1:100 vol/vol)). Bars indicate mean + SD. N= 5 newborns, 7 adults. ** p<0.05; ** p<0.01;* (blue stars: newborn vs. vehicle; red stars: adult vs. vehicle).

*
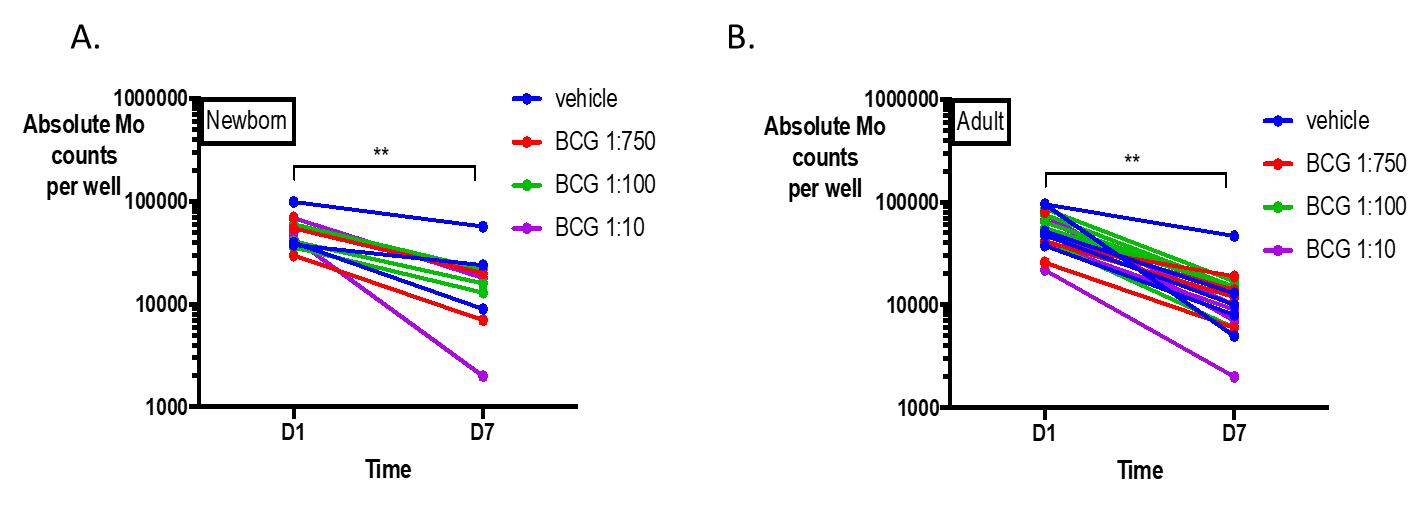
***Supplementary Figure 3. Culture with BCG leads to decrease in monocyte (Mo) viability across time.** Human newborn and adult monocytes were cultured *in vitro* with BCG as described in Methods. Absolute newborn (A) and adult (B) monocyte counts are depicted at Days 1 (D1) and 7 (D7) of culture based on starting monocyte count of 100,000 Mos/well on Day 0 (D0), as assessed after detachment with ethylenediaminetetraacetic acid (EDTA) and trypan blue exclusion under microscopy. Each color represents a different treatment condition, and each line of the same color represents a different study participant. *** p<0.01* by paired t-test. N= 3 newborns, 5 adults.


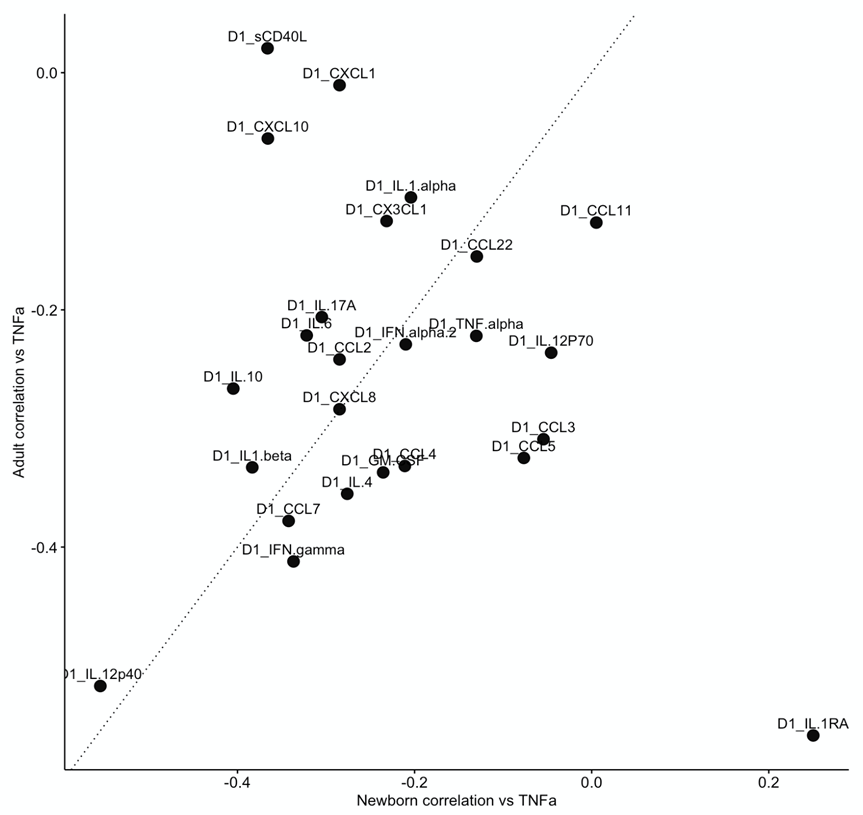


**Supplementary Figure 4. Relationship between newborn and adult monocyte correlation coefficients for Day 1 cytokines vs. Day 7 TNF.** Scatterplot demonstrating the relationship between newborn (x-axis) and adult (y-axis) correlation coefficients for D1 cytokines/chemokines vs. D7 TNFα. The dotted line represents x=y.

**Supplementary Table 1.** Summative statistics (minimum, maximum, median, interquartile range) of absolute cytokine/chemokine concentrations (pg/mL) on Day 1 of monocyte culture.

| **Day 1 Cytokines/**  **Chemokines** | **Newborn** | | | | **Adult** | | | |
| --- | --- | --- | --- | --- | --- | --- | --- | --- |
|  | **Min** | **Max** | **Median** | **IQR** | **Min** | **Max** | **Median** | **IQR** |
| **CCL11** | 4.0 |  |  |  | 4.0 |  |  |  |
| Veh | 0.78 | 147 | 0.78 | 12.97 | 0.78 | 75 | 0.78 | 22.665 |
| BCG low | 0.78 | 141 | 88.73 | 86.96 | 0.78 | 132 | 11.36 | 69.79 |
| BCG high | 0.78 | 191 | 130 | 60 | 36.47 | 161 | 122 | 39.635 |
| **GM-CSF** | 7.5 |  |  |  | 7.5 |  |  |  |
| Veh | 4.48 | 201 | 48.13 | 137.18 | 1.23 | 140 | 44.23 | 64.61 |
| BCG low | 58.98 | 2074 | 293 | 488 | 11.32 | 160 | 54.2 | 53.415 |
| BCG high | 1.23 | 4095 | 3840 | 3354 | 243 | 1062 | 652 | 465.5 |
| **CX3CL1** | 22.7 |  |  |  | 22.7 |  |  |  |
| Veh | 0.89 | 188 | 74.09 | 150.08 | 0.89 | 558 | 65.33 | 276.92 |
| BCG low | 105 | 330 | 293 | 70 | 0.89 | 544 | 148 | 317.055 |
| BCG high | 0.89 | 350 | 320 | 35 | 178 | 675 | 249 | 262.5 |
| **IFNα2** | 2.9 |  |  |  | 2.9 |  |  |  |
| Veh | 3.22 | 113 | 39.11 | 43.16 | 1.46 | 99.07 | 26.63 | 24.025 |
| BCG low | 33.92 | 167 | 124 | 52.22 | 1.46 | 64.12 | 26.24 | 25.91 |
| BCG high | 1.46 | 232 | 175 | 45 | 88.88 | 137 | 127 | 12.5 |
| **IFNγ** | 0.8 |  |  |  | 0.8 |  |  |  |
| Veh | 0.2 | 28.25 | 6.82 | 22.93 | 0.2 | 32.18 | 10.3 | 12.64 |
| BCG low | 9.69 | 35.65 | 26.19 | 15.34 | 2.99 | 25.68 | 11.54 | 5.23 |
| BCG high | 0.2 | 51.74 | 46.1 | 11.96 | 22.17 | 56.87 | 37.28 | 21.45 |
| **CXCL1** | 9.9 |  |  |  | 9.9 |  |  |  |
| Veh | 467 | 21093.88 | 3232 | 2955 | 237 | 21093.88 | 2763 | 5581.5 |
| BCG low | 21093.88 | 21093.88 | 21093.88 | 0 | 1069 | 21093.88 | 2481 | 11229.44 |
| BCG high | 5.21 | 21093.88 | 21093.88 | 0 | 21093.88 | 21093.88 | 21093.88 | 0 |
| **IL-10** | 1.1 |  |  |  | 1.1 |  |  |  |
| Veh | 1.03 | 4.3 | 1.03 | 0 | 1.03 | 2.75 | 1.03 | 0.69 |
| BCG low | 1.03 | 49.4 | 23.01 | 14.37 | 1.03 | 26.52 | 1.03 | 3.9 |
| BCG high | 1.03 | 581 | 142 | 331.42 | 2.26 | 503 | 36.31 | 54.8 |
| **CCL7** | 3.8 |  |  |  | 3.8 |  |  |  |
| Veh | 26.44 | 22609.02 | 1018 | 1577 | 26.44 | 22609.02 | 3308 | 3786.78 |
| BCG low | 906 | 7518 | 2194 | 3343 | 26.44 | 7664 | 1927 | 1692 |
| BCG high | 26.44 | 7870 | 2695 | 4807 | 3049 | 22609.02 | 4715 | 11066.01 |
| **IL-12p40** | 7.4 |  |  |  | 7.4 |  |  |  |
| Veh | 0.42 | 0.42 | 0.42 | 0 | 0.42 | 0.42 | 0.42 | 0 |
| BCG low | 0.42 | 0.42 | 0.42 | 0 | 0.42 | 0.42 | 0.42 | 0 |
| BCG high | 0.42 | 108 | 47.49 | 25.42 | 0.42 | 66.98 | 0.42 | 0 |
| **CCL22** | 3.6 |  |  |  | 3.6 |  |  |  |
| Veh | 1236 | 10090 | 2218 | 1384 | 563 | 7013 | 4631 | 5105.5 |
| BCG low | 785 | 5815 | 1703 | 1694 | 904 | 2906 | 1434 | 562.5 |
| BCG high | 1.07 | 4568 | 1259 | 382 | 1658 | 3955 | 2898 | 697.5 |
| **IL-12p70** | 0.6 |  |  |  | 0.6 |  |  |  |
| Veh | 0.2 | 2.26 | 0.2 | 1.32 | 0.2 | 9.01 | 0.2 | 3.845 |
| BCG low | 0.2 | 10.02 | 5.84 | 3 | 0.2 | 16.49 | 0.2 | 1.43 |
| BCG high | 0.2 | 14.82 | 12.46 | 10.46 | 0.81 | 19.92 | 5.84 | 2.93 |
| **sCD40L** | 5.1 |  |  |  | 5.1 |  |  |  |
| Veh | 0.2 | 14.26 | 9.36 | 1.46 | 0.2 | 13.63 | 9.36 | 9.605 |
| BCG low | 4.65 | 18.12 | 4.91 | 4.45 | 0.2 | 12.69 | 0.2 | 3.715 |
| BCG high | 0.2 | 29.14 | 15.53 | 21.69 | 0.2 | 11.77 | 4.4 | 6.36 |
| **IL-17A** | 0.7 |  |  |  | 0.7 |  |  |  |
| Veh | 0.38 | 6.08 | 3.1 | 1.3 | 0.38 | 6.82 | 4.26 | 3.69 |
| BCG low | 2.18 | 7.51 | 7.13 | 4.2 | 1.96 | 7.35 | 3.35 | 2.045 |
| BCG high | 0.38 | 12.2 | 7.58 | 2.37 | 5.22 | 8.21 | 6.52 | 1.04 |
| **IL-1RA** | 8.3 |  |  |  | 8.3 |  |  |  |
| Veh | 425 | 14157 | 2617 | 2965 | 83.94 | 5220 | 2462 | 2682.5 |
| BCG low | 405 | 10452 | 2624 | 5818 | 1001 | 5431 | 1519 | 920 |
| BCG high | 268 | 6341 | 1369 | 1936 | 2264 | 5355 | 2616 | 1704 |
| **IL-1α** | 9.4 |  |  |  | 9.4 |  |  |  |
| Veh | 0.25 | 126 | 14.59 | 71.12 | 0.25 | 23.02 | 0.5 | 10.705 |
| BCG low | 80.49 | 790 | 256 | 335 | 0.25 | 514 | 4.6 | 36.155 |
| BCG high | 1194 | 3279 | 1773 | 970 | 28.86 | 2433 | 242 | 404 |
| **IL-1β** | 0.8 |  |  |  | 0.8 |  |  |  |
| Veh | 0.2 | 32.05 | 0.2 | 3.51 | 0.2 | 6.26 | 0.2 | 2.56 |
| BCG low | 14.37 | 173 | 103 | 43.65 | 0.2 | 176 | 3.91 | 8.68 |
| BCG high | 0.2 | 2629 | 555 | 1797 | 89.48 | 2382 | 208 | 354.755 |
| **IL-4** | 4.5 |  |  |  | 4.5 |  |  |  |
| Veh | 6.21 | 107 | 23.49 | 32.15 | 3.08 | 29.02 | 8.22 | 4.785 |
| BCG low | 27.75 | 64.82 | 49.5 | 15.54 | 1.95 | 31.63 | 11.76 | 10.215 |
| BCG high | 0.55 | 113 | 70.09 | 19.45 | 8.4 | 84.75 | 44.47 | 23.075 |
| **IL-6** | 0.9 |  |  |  | 0.9 |  |  |  |
| Veh | 1.78 | 180 | 36.3 | 46.26 | 1.78 | 141 | 10.7 | 28.26 |
| BCG low | 185 | 8143 | 3447 | 4115 | 30.58 | 2714 | 80.95 | 128.1 |
| BCG high | 1.78 | 30405.7 | 30405.7 | 15398.7 | 563 | 14342 | 8016 | 7578.5 |
| **CXCL8** | 0.4 |  |  |  | 0.4 |  |  |  |
| Veh | 1703 | 22329.7 | 22329.7 | 16134.7 | 302 | 22329.7 | 22329.7 | 19079.7 |
| BCG low | 22329.7 | 22329.7 | 22329.7 | 0 | 4532 | 22329.7 | 22329.7 | 6516.85 |
| BCG high | 65.29 | 22329.7 | 22329.7 | 0 | 22329.7 | 22329.7 | 22329.7 | 0 |
| **CXCL10** | 8.6 |  |  |  | 8.6 |  |  |  |
| Veh | 0.43 | 109 | 0.43 | 0 | 26.88 | 422 | 111 | 85.19 |
| BCG low | 0.43 | 124 | 0.43 | 0 | 0.43 | 297 | 0.43 | 73.785 |
| BCG high | 0.43 | 826 | 122 | 117 | 11.46 | 125 | 52.43 | 47.345 |
| **CCL2** | 1.9 |  |  |  | 1.9 |  |  |  |
| Veh | 1000 | 21257.88 | 21257.88 | 13767.88 | 576 | 21257.88 | 7846 | 18845.88 |
| BCG low | 21257.88 | 21257.88 | 21257.88 | 0 | 1677 | 21257.88 | 9207 | 13654.88 |
| BCG high | 174 | 21257.88 | 21257.88 | 0 | 6097 | 21257.88 | 21257.88 | 5661.44 |
| **CCL3** | 2.9 |  |  |  | 2.9 |  |  |  |
| Veh | 32.51 | 16790.12 | 99.06 | 6501.44 | 2.98 | 2636 | 136 | 826.49 |
| BCG low | 169 | 16790.12 | 16790.12 | 14716.12 | 49.01 | 5857 | 189 | 1015.5 |
| BCG high | 2.98 | 16790.12 | 16790.12 | 0 | 1587 | 16790.12 | 6984 | 7976.06 |
| **CCL4** | 3.0 |  |  |  | 3.0 |  |  |  |
| Veh | 78.82 | 3748 | 268 | 3167 | 0.39 | 1851 | 296 | 728.565 |
| BCG low | 400 | 8823 | 3833 | 2210 | 192 | 2700 | 643 | 779 |
| BCG high | 0.39 | 11561 | 9601 | 8452 | 1129 | 8848 | 6064 | 4356.5 |
| **CCL5** | 1.2 |  |  |  | 1.2 |  |  |  |
| Veh | 590 | 23274.36 | 6106 | 7504 | 761 | 9542 | 1652 | 2920.5 |
| BCG low | 1722 | 10577 | 7254 | 8132 | 594 | 8648 | 1085 | 3724.5 |
| BCG high | 11.22 | 23274.36 | 6533 | 6587 | 842 | 10741 | 4903 | 4341 |
| **TNFα** | 0.7 |  |  |  | 0.7 |  |  |  |
| Veh | 36 | 1364 | 99.28 | 844.06 | 16.48 | 819 | 216 | 258.77 |
| BCG low | 94.41 | 7047 | 1947 | 1976 | 55.66 | 1083 | 265 | 267.5 |
| BCG high | 4.17 | 8869 | 6746 | 5968 | 750 | 6717 | 2535 | 1918 |

**Supplementary Table 2**. Summative statistics (minimum, maximum, median, interquartile range) of absolute cytokine/chemokine concentrations (pg/mL) on Day 7 of monocyte culture.

| **Day 7 Cytokines/**  **Chemokines** | **Newborn** | | | | **Adult** | | | |
| --- | --- | --- | --- | --- | --- | --- | --- | --- |
|  | **Min** | **Max** | **Median** | **IQR** | **Min** | **Max** | **Median** | **IQR** |
| **CCL11** | 4.0 |  |  |  | 4.0 |  |  |  |
| Veh+LPS | 0.78 | 145 | 13.75 | 120.22 | 8.8 | 127 | 84.28 | 44.1 |
| BCG low+LPS | 0.78 | 148 | 22.23 | 116.22 | 0.78 | 129 | 54.46 | 88.65 |
| BCG high+LPS | 0.78 | 101 | 47.32 | 40.29 | 36.47 | 162 | 102 | 51.005 |
| **GM-CSF** | 7.5 |  |  |  | 7.5 |  |  |  |
| Veh+LPS | 51.28 | 4327 | 379 | 1014 | 322 | 3390 | 1867 | 895.5 |
| BCG low+LPS | 222 | 2710 | 235 | 2343 | 958 | 2643 | 1560 | 733.5 |
| BCG high+LPS | 186 | 4031 | 682 | 567 | 740 | 5337 | 3125 | 2325.5 |
| **CX3CL1** | 22.7 |  |  |  | 22.7 |  |  |  |
| Veh+LPS | 0.89 | 254 | 161 | 187.09 | 59.13 | 555 | 213 | 81.5 |
| BCG low+LPS | 0.89 | 205 | 122 | 182.11 | 79.63 | 609 | 156 | 97.5 |
| BCG high+LPS | 37.99 | 216 | 139 | 90 | 141 | 519 | 188 | 68 |
| **IFNα2** | 2.9 |  |  |  | 2.9 |  |  |  |
| Veh+LPS | 8.72 | 125 | 103 | 84.08 | 111 | 164 | 120 | 19 |
| BCG low+LPS | 38.23 | 129 | 84.43 | 68.77 | 71.43 | 233 | 118 | 22 |
| BCG high+LPS | 22.5 | 125 | 76.78 | 50.21 | 115 | 171 | 133 | 32 |
| **IFNγ** | 0.8 |  |  |  | 0.8 |  |  |  |
| Veh+LPS | 1.54 | 54.3 | 25.68 | 19.67 | 17.82 | 55.73 | 23.16 | 13.045 |
| BCG low+LPS | 2.99 | 50.04 | 26.19 | 23.26 | 17.58 | 66.71 | 31.91 | 6.54 |
| BCG high+LPS | 13.48 | 33.51 | 15.5 | 8.73 | 19.72 | 58.6 | 33.51 | 11.71 |
| **CXCL1** | 9.9 |  |  |  | 9.9 |  |  |  |
| Veh+LPS | 369 | 21093.88 | 2361 | 18959.88 | 1596 | 21093.88 | 2486 | 3183.5 |
| BCG low+LPS | 1206 | 9985 | 4443 | 7021 | 1028 | 21093.88 | 3030 | 4478.5 |
| BCG high+LPS | 1023 | 21093.88 | 4335 | 18570.88 | 1669 | 21093.88 | 21093.88 | 8132.94 |
| **IL-10** | 1.1 |  |  |  | 1.1 |  |  |  |
| Veh+LPS | 28.67 | 19658.92 | 396 | 935 | 21.2 | 3276 | 459 | 1473 |
| BCG low+LPS | 74.03 | 19658.92 | 211 | 28 | 48.68 | 7036 | 1062 | 1849.5 |
| BCG high+LPS | 1.03 | 6998 | 60.49 | 120.67 | 29.45 | 3430 | 769 | 935.5 |
| **CCL7** | 3.8 |  |  |  | 3.8 |  |  |  |
| Veh+LPS | 26.44 | 3061 | 26.44 | 573.56 | 26.44 | 3212 | 26.44 | 0 |
| BCG low+LPS | 26.44 | 829 | 26.44 | 0 | 26.44 | 2901 | 26.44 | 13.22 |
| BCG high+LPS | 26.44 | 1102 | 26.44 | 0 | 26.44 | 5698 | 26.44 | 0 |
| **IL-12p40** | 7.4 |  |  |  | 7.4 |  |  |  |
| Veh+LPS | 0.42 | 1363 | 263 | 707.55 | 0.42 | 2160 | 513 | 866.255 |
| BCG low+LPS | 0.42 | 483 | 57.92 | 290.58 | 0.84 | 720 | 453 | 394.41 |
| BCG high+LPS | 0.42 | 120 | 0.42 | 62.84 | 0.42 | 819 | 225 | 585.835 |
| **CCL22** | 3.6 |  |  |  | 3.6 |  |  |  |
| Veh+LPS | 1.07 | 9854 | 395 | 6979.93 | 126 | 2986 | 538 | 923 |
| BCG low+LPS | 1.07 | 3764 | 751 | 2046.75 | 183 | 6205 | 874 | 905 |
| BCG high+LPS | 1.07 | 2594 | 41.41 | 1106.4 | 597 | 23432.42 | 1119 | 1509 |
| **IL-12p70** | 0.6 |  |  |  | 0.6 |  |  |  |
| Veh+LPS | 0.2 | 47.55 | 4.68 | 19.37 | 4.68 | 134 | 59.23 | 83.545 |
| BCG low+LPS | 0.2 | 13 | 2.72 | 11.55 | 6.76 | 46.26 | 29.08 | 22.46 |
| BCG high+LPS | 0.2 | 5.55 | 2.15 | 1.95 | 1.93 | 51.63 | 12.46 | 11.285 |
| **sCD40L** | 5.1 |  |  |  | 5.1 |  |  |  |
| Veh+LPS | 0.2 | 26.09 | 0.2 | 3.7 | 0.2 | 7.34 | 0.2 | 3.625 |
| BCG low+LPS | 0.2 | 5.43 | 2.3 | 3.95 | 0.2 | 14.89 | 4.4 | 5.01 |
| BCG high+LPS | 0.2 | 12.07 | 0.2 | 9.75 | 0.2 | 12.38 | 3.9 | 7.04 |
| **IL-17A** | 0.7 |  |  |  | 0.7 |  |  |  |
| Veh+LPS | 1.1 | 7.43 | 3.35 | 5.27 | 3.6 | 10.37 | 5.22 | 1.655 |
| BCG low+LPS | 0.38 | 7.51 | 3.1 | 4.67 | 3.6 | 8.84 | 5.43 | 2.18 |
| BCG high+LPS | 0.78 | 8.76 | 2.12 | 6.52 | 3.29 | 10.37 | 6.08 | 2.385 |
| **IL-1RA** | 8.3 |  |  |  | 8.3 |  |  |  |
| Veh+LPS | 0.5 | 5652 | 791 | 1694.51 | 428 | 5831 | 998 | 731.5 |
| BCG low+LPS | 0.5 | 4655 | 1409 | 2468 | 799 | 5055 | 1874 | 1369.5 |
| BCG high+LPS | 0.5 | 2553 | 163 | 1279.98 | 666 | 7712 | 2615 | 1929 |
| **IL-1α** | 9.4 |  |  |  | 9.4 |  |  |  |
| Veh+LPS | 0.25 | 570 | 107 | 139.95 | 34.82 | 168 | 69.85 | 56.81 |
| BCG low+LPS | 78.22 | 304 | 158 | 113.78 | 1.61 | 357 | 75.94 | 81.8 |
| BCG high+LPS | 72.9 | 1452 | 564 | 1103.21 | 21.58 | 888 | 235 | 180 |
| **IL-1β** | 0.8 |  |  |  | 0.8 |  |  |  |
| Veh+LPS | 0.2 | 28.17 | 0.76 | 2.49 | 0.2 | 33.59 | 14.8 | 19.15 |
| BCG low+LPS | 0.2 | 15.39 | 0.2 | 13.88 | 0.39 | 29.88 | 12.18 | 17.58 |
| BCG high+LPS | 0.2 | 119 | 5.25 | 6.06 | 8.37 | 75.11 | 32.05 | 27.82 |
| **IL-4** | 4.5 |  |  |  | 4.5 |  |  |  |
| Veh+LPS | 0.55 | 75.48 | 13.3 | 18.03 | 6.21 | 51.48 | 24.97 | 15.29 |
| BCG low+LPS | 2.04 | 11.33 | 5.9 | 9.03 | 7.52 | 48.32 | 12.63 | 5.24 |
| BCG high+LPS | 1.12 | 31.63 | 8.22 | 6.94 | 9.33 | 38.56 | 17.16 | 9.64 |
| **IL-6** | 0.9 |  |  |  | 0.9 |  |  |  |
| Veh+LPS | 349 | 6834 | 2006 | 4510 | 1588 | 11638 | 5031 | 2213 |
| BCG low+LPS | 508 | 8640 | 867 | 1730 | 2425 | 7942 | 3725 | 1352.5 |
| BCG high+LPS | 747 | 13324 | 2076 | 2597 | 2727 | 11728 | 6775 | 4882.5 |
| **CXCL8** | 0.4 |  |  |  | 0.4 |  |  |  |
| Veh+LPS | 2345 | 22329.7 | 11120 | 12205.7 | 10009 | 22329.7 | 22329.7 | 12028.7 |
| BCG low+LPS | 7794 | 22329.7 | 22329.7 | 13924.7 | 5469 | 22329.7 | 22329.7 | 6316.85 |
| BCG high+LPS | 7584 | 22329.7 | 22329.7 | 0 | 10435 | 22329.7 | 22329.7 | 0 |
| **CXCL10** | 8.6 |  |  |  | 8.6 |  |  |  |
| Veh+LPS | 0.43 | 21350.76 | 160 | 436.9 | 537 | 10327 | 1591 | 595 |
| BCG low+LPS | 47.8 | 3964 | 273 | 244 | 683 | 21350.76 | 1579 | 2873.5 |
| BCG high+LPS | 0.43 | 6629 | 323 | 758 | 605 | 21350.76 | 1568 | 2689.5 |
| **CCL2** | 1.9 |  |  |  | 1.9 |  |  |  |
| Veh+LPS | 415 | 21257.88 | 4466 | 19793.88 | 1439 | 21257.88 | 8441 | 8993.44 |
| BCG low+LPS | 1099 | 21257.88 | 10314 | 11114.88 | 1346 | 21257.88 | 4925 | 9762.94 |
| BCG high+LPS | 1942 | 21257.88 | 21257.88 | 0 | 1290 | 21257.88 | 9435 | 15474.88 |
| **CCL3** | 2.9 |  |  |  | 2.9 |  |  |  |
| Veh+LPS | 361 | 16790.12 | 3088 | 14599.12 | 3795 | 16790.12 | 7718 | 9547.62 |
| BCG low+LPS | 1195 | 16790.12 | 16790.12 | 14990.12 | 8395.06 | 16790.12 | 16790.12 | 0 |
| BCG high+LPS | 1059 | 16790.12 | 1593 | 5305 | 2615 | 16790.12 | 16790.12 | 11182.12 |
| **CCL4** | 3.0 |  |  |  | 3.0 |  |  |  |
| Veh+LPS | 507 | 28007.84 | 4489 | 26017.84 | 3354 | 28007.84 | 10423 | 11952.92 |
| BCG low+LPS | 1077 | 28007.84 | 7035 | 26496.84 | 6848 | 28007.84 | 14003.92 | 19960.84 |
| BCG high+LPS | 745 | 28007.84 | 1424 | 26995.84 | 5295 | 28007.84 | 28007.84 | 7781.92 |
| **CCL5** | 1.2 |  |  |  | 1.2 |  |  |  |
| Veh+LPS | 11.22 | 23274.36 | 11.22 | 2997.78 | 146 | 7908 | 1302 | 2681 |
| BCG low+LPS | 11.22 | 4533 | 65.39 | 68.41 | 122 | 7436 | 1454 | 2768.5 |
| BCG high+LPS | 11.22 | 7060 | 11.22 | 494.78 | 1088 | 7830 | 2307 | 1274.5 |
| **TNFα** | 0.7 |  |  |  | 0.7 |  |  |  |
| Veh+LPS | 513 | 22512.04 | 9895 | 18633.04 | 5272 | 22512.04 | 22512.04 | 12904.04 |
| BCG low+LPS | 1129 | 22512.04 | 10013 | 20990.04 | 9189 | 22512.04 | 22512.04 | 5913.02 |
| BCG high+LPS | 1064 | 10734 | 2469 | 1859 | 7088 | 22512.04 | 22512.04 | 13044.04 |
